# DEVELOPING A GERIATRIC ONCOLOGY FELLOWSHIP SUBSPECIALTY TRACK FOR ADVANCED PRACTICE PROVIDERS

**DOI:** 10.1093/geroni/igad104.2533

**Published:** 2023-12-21

**Authors:** Sincere McMillan, Beatriz Korc-Grodzicki, Amelia Cataldo, Nicole Zakak, Kelly Haviland

**Affiliations:** Memorial Sloan Kettering Cancer Center, New York City, New York, United States; Memorial Sloan Kettering Cancer Center, New York City, New York, United States; Memorial Sloan Kettering Cancer Center, New York City, New York, United States; Memorial Sloan Kettering Cancer Center, New York City, New York, United States; Memorial Sloan Kettering Cancer Center, New York City, New York, United States

## Abstract

Although a relatively young subspeciality, geriatric oncology is a fast-growing discipline. As the population ages, clinicians skilled in geriatric oncology principles and management are needed to care for an increasingly complex older adult population. Educating Advanced Practice Providers (APP) in geriatric oncology may offer innovative ways of increasing access to care, supporting patient health, and improving quality of life. The need for trained clinicians represented a unique opportunity for the development of a geriatric oncology focused APP fellowship subspecialty track at our institution. In 2019, with support from the Samuels Foundation, an electronic geriatric oncology needs assessment was completed among the APPs at our institution. Data from this needs assessment and from a thorough literature review were used to develop the geriatric oncology fellowship track curriculum. With support from APP leadership at our institution, the fellowship track launched in September, 2021. It is a 12-month postgraduate academic program that focuses on geriatric assessment, risk stratification prior to cancer directed therapies, management of geriatric syndromes, and geriatric co-management. Additionally in 2023, the fellowship track will include an elective in a long-term care facility. We believe this unique elective rotation will be a strong addition to the clinical experience of the fellow. Our current program has one position per fellowship year with the goal of increasing the number of positions in the future. This is a major step forward in the education of APPs in the care of older adults with cancer that we hope will be replicated across the country.

